# Supporting men through their transition to fatherhood with messages delivered to their smartphones: a feasibility study of SMS4dads

**DOI:** 10.1186/s12889-017-4978-0

**Published:** 2017-12-13

**Authors:** Richard Fletcher, Francis Kay-Lambkin, Chris May, Christopher Oldmeadow, John Attia, Lucy Leigh

**Affiliations:** 1 Faculty of Health and Medicine, The University of Newcastle, Callaghan, NSW 2308 Australia; 2 Hunter Medical Research Institute, LOT 1 kookaburra circuit, New Lambton Heights, NSW 2305 Australia

**Keywords:** Fathers, Fatherhood, Digital interventions, Antenatal, Postnatal, Distress, Mental health

## Abstract

**Background:**

The transition to parenthood can be a challenging time, in which both mothers and fathers experience increased risk of distress and depression. Mothers are more likely than fathers to engage with services and have their mental health monitored and attended to during the perinatal period. The present study aimed to explore whether smartphone technology could be used to address fathers’ needs across their transition to fatherhood.

**Methods:**

A corpus of messages, including linked information and mood tracking software, was designed to support and enhance paternal relationships with their babies, their partners and themselves across the perinatal period. Messages were sent to project participants (*N* = 520) from 12-weeks’ gestation to 24-weeks after birth.

**Results:**

Of those fathers enrolled (*N* = 520), 21.5% scored >13 on K6 and completion rate (85%) was similar between these and other fathers. Most fathers (63.1%) clicked at least one link and responses were received for 20.5% of mood tracker questions. The probability of reporting worse mood scores decreased over time. Fathers completing post study surveys (*N* = 101) reported that messages helped them in their experience of becoming a new dad (92.8%), as well as helping them develop a strong relationship with their new child (54.9%), and in their relationship with their partner (79%).

**Conclusions:**

The present study has demonstrated that it is both feasible and acceptable to support new fathers with SMS4dads, a relationship-focused messaging system designed to be delivered to smartphones across fathers’ transition to parenthood.

## Background

The transition to parenthood for both mothers and fathers carries expectations of joy and wonder [1, 2]. However the reality of childbirth, the demands of the new baby, and challenge in reconfiguring their relationships and identity can bring parents exhaustion, confusion and stress, leading to anxiety and depression. Postnatal depression in mothers is characterised by feelings of worthlessness, decreased interest in pleasure, dysphoria, anxiety, inability to make decisions and impaired infant care, creating a suboptimal environment for newborns [3]. Maternal depression, which affects up to 25% of women during pregnancy and 10–15% during the first postnatal year has been shown to double the rate of emotional–behavioural difficulties in children at 4 years of age when compared to children of symptom free women [4].

Mood disorders among fathers have been less well studied but the emerging evidence suggests parallels in the extent and impact of paternal perinatal depression and anxiety. A meta-analysis of 43 studies of fathers’ mental health from North America, Europe, England and Australia found an average rate of 10.4% for paternal depression between the first trimester and 1 year postpartum [5]. In similar fashion, a review of studies examining anxiety among expectant and new fathers found rates up to 16.0% during the prenatal period and up to 18.0% during the postnatal period, although there was wide variation between studies [6]. Paternal depression may also influence a fathers’ parenting and therefore the wellbeing of his infant into the future. Depressed fathers in the USA, for example, were 4 times more likely to spank their one year old babies and less than half as likely to read to them as non-depressed fathers [7]. Studies following infants whose fathers showed signs of postnatal depression through to childhood show that these infants are three times more likely to exhibit behaviour problems as a pre-schooler [8] and twice as likely to receive a psychiatric diagnosis by 7 years of age [9].

A major difference in the response to maternal and paternal mood disorders is the provision of screening and referral for treatment. To identify mothers at risk of mental illness in Australia, health services have instituted screening processes to identify maternal depression and make referrals [10]. While the most widely used screening instrument for women, the Edinburgh Postnatal Depression Scale, has been validated for men [11], there are no reports in the literature of services designed to assess and refer fathers for mood disorders in the perinatal period.

The lack of appropriate services for addressing new fathers’ mental health can be attributed to a combination of factors including fathers’ occupation patterns, the historical focus of birthing services on maternal health and staff and community attitudes. Work demands, for example, prevent most fathers participating in the antenatal and postnatal clinic visits with their partner [12]. Fathers attending the ultrasound, antenatal preparation classes or the birth report that in many cases they feel ignored by the professionals involved and have few opportunities to raise their concerns [13, 14]. Intergenerational patterns of mothers’ exclusive role in infant care may leave fathers with little experience caring for newborns. Their return to full time work soon after the birth may cement a fathers’ lack of confidence in handling their babies, leaving them dependent on the mother’s guidance [12, 15]. Offering parenting education to new fathers has not proved to be a fruitful way to provide support. Efforts over many years to engage fathers in early intervention programs have had little success and fathers are badged as ‘hard to reach’ [16, 17].

The widespread adoption of mobile technology may present an opportunity to deliver information and support to fathers. Mobile phone ownership among Australian adults is high (81%) and increasing and those aged 18 to 44 (which spans the median age (33) for a father’s first child) are the most active users of communications apps [18–20]. Mobile phone messages are inexpensive and the universal access to mobile phone networks means that texts to mobile phones may provide access to hard to reach populations such as new fathers across metropolitan, rural and remote populations even though coverage across regions is uneven.

Text messaging to mothers has been shown to influence health related knowledge and beliefs. Text4baby, a US based program sending SMS messages to mothers through the perinatal period has found that 75% of users reported text messaging had given them important medical information. A randomised control trial involving 90 users found that text messaging was associated with positive alterations in targeted health beliefs [21]. Health behaviours relating to smoking, weight loss, physical activity and the management of diabetes have also shown improvement in response to text messaging [22, 23]. Text messaging could therefore provide a novel avenue for delivering support and parenting related information to fathers during their transition to fatherhood.

The aim of this study was to examine the feasibility and acceptability of delivering web-based text as a depression prevention program to men in their transition to fatherhood. Although fathers in Australia comprise a range of language and cultural groups the present study did not attempt to map the characteristics of participants at enrolment in any detail. We recognise that specific strategies would be required to engage particular populations, and in a separate study we successfully engage young Aboriginal fathers [24], however our recruitment strategies in this feasibility study were broadly aimed through universal perinatal services and Australia-wide social media. We were interested in comparing the ability of health professionals to recruit fathers with self-referral via social media. We also wished to know if fathers would be willing to enrol and to remain in the program until their infant reached six months and whether the links to other online resources would be utilised. Participants’ responses to the Mood Tracker were seen as mapping the fathers’ level of concern as was the number requiring telephone contact. Post program surveys were designed to provide a quantitative evaluation of the program’s acceptability by completing fathers and open comments were sought to identify noteworthy aspects of the program as seen by the participants. The influence of fathers’ level of distress, age and socioeconomic background on their engagement with SMS4dads was of particular interest. We wished to discover if these factors would influence fathers’ tendency to enrol, their willingness to stay engaged and utilize the links, and also their responses to the Mood Tracker.

## Methods

### Development of SMS4dads text messages

The development of the text messages for SMS4dads occurred through an iterative feasibility and acceptability process described in detail elsewhere [25, 26]. Briefly, initial messages were developed by experienced practitioners and assigned to three a priori research-based categories of information and support for new fathers: (1) the father-infant connection, (2) fathers’ support for the mother, and (3) fathers’ self-care [27–30]. The draft messages which were tailored to appeal to men transitioning to fatherhood were then reviewed by an expert panel and adapted accordingly. The final set of messages consisted of 184 texts and 17 Mood Tracker messages.

The study was approved by the Hunter New England Health Human Research Ethics.

Committee (Ref: 15/09/16/4.07) and the University of Newcastle Human Research Ethics.

Committee (Ref: H-2016-0037).

### Participants and recruitment

The study enrolled men expecting a baby in the next six months, or fathers with infants under three months of age. Participants were required to have a mobile smart phone and to be able to read English. They were not compensated for taking part in the study. Recruitment occurred through flyers and posters at hospital-based parenting education, antenatal clinics, and ultrasound services and at postnatal services such as Neonatal Intensive Care Unit (NICU) and home visits by Child and Family Health Nurses. Clinical staff were informed of the project through face-to-face education sessions (Hunter Valley sites only) and through professional meetings and conferences throughout Australia. Social media sites were also utilised to alert professionals and parents of the availability of SMS4dads. Mothers’ Facebook sites covering specific regions with substantial memberships (some of more than 10,000) were contacted to place posts describing the research and asking for participants and a national online mothers’ site was contracted to include SMS4dads banners and posts promoting the research.

Men enrolled online via phone or computer, completed an informed consent form, provided demographic information including their age and their child’s expected or actual date of birth and indicated where they had heard of SMS4dads. Participants also completed the Kessler K6, a brief six-question screening tool for mental illness in general populations [31]. Men who consented to having their partner invited were sent a maternal recruitment package asking permission to conduct a telephone interview with their partner at the conclusion of the study.

### The SMS4dads intervention

The intervention consisted of a set of 184 brief (160 characters or less) text messages delivered on a four week cycle with frequencies of five, four, three, and two per week followed by a new cycle of five, four etc. Message content addressed his relationship with his baby (*n* = 72), his relationship with and support of the baby’s mother (*n* = 61), and his own self-care (*n* = 49) (See example messages in Table 1). The SMS4dads project also connected the new fathers and fathers-to-be, through links in the SMS messages, to a variety of non-profit and government-based online resources. Fifty-two resource links were included in (34%) of the messages with some links being used for more than one message. The message schedule was tailored to gestation and infant’s age; therefore not all messages and links were received by all the fathers. Moodtracker questions (*n* = 17) were sent every three weeks while instructions for opting out were included in 26% of all texts.

**Table 1:** Message examples

Week	Example Text	Content Category
1	Talk to your partner about staying home in the early months. Are there ways you can get more leave?[Txt STOP to OptOut] http://www.fairwork.gov.au/leave/maternity-and-parental-leave/paid-parental-leave^a^	Father-partner
2	Babies come with personality dad. Getting to know my personality can make being my dad much more rewarding for you.[Txt STOP to OptOut]	Father-baby
3	Good food, good sleep and lots of exercise can really help a lot at this time. [Txt STOP to OptOut] http://raisingchildren.net.au/looking_after_yourself/looking_after_yourself.html^a^	Father self-care
4	How are you going with picking names? Trying to imagine your baby’s personality is one way to start the bonding process.[Txt STOP to OptOut]	Father-baby
5	Doing it alone? This is the time to ask for support. Let family and friends know how they can help. http://raisingchildren.net.au/articles/grownups_services_and_support_nutshell.html^a^	Father self-care

^a^A brief version of the URL is included in the message which keeps the SMS within 160 characters and allows the tracking of the clicked links (although what a father does once he lands on the web page is not recorded)

An interactive Mood Tracker text asked participants to submit mood information by selecting one of five one-click options (‘awesome’, ‘cool’, ‘OK’, ‘shaky’ or ‘bad’). Four questions were repeated on a three-weekly cycle: “How are you travelling?” (understood as ‘How are you feeling?’), “How are you coping with things today?”, “Is fatherhood working out as you thought it would?”, and “How is your stress level?” For responses other than ‘bad’ an appropriate encouraging return message was sent. If the participant responded ‘bad’, an escalation process was put in place offering a phone call from a national help-line for perinatal mental health support.

### Uptake, user engagement and acceptability

The uptake of the intervention was evaluated by data collected on recruitment rates for locations within Australia, and the reported source of information on SMS4dads. The number of weeks receiving messages, use of the ‘STOP’ text to discontinue, use of embedded website links, and responses to the Mood Tracker were recorded by the software. A post-intervention structured phone interview comprising 11 Likert-scale questions asked participants to judge the acceptability and usefulness of the messages and the SMS process. A similar interview for mothers comprising nine items asked about their partner’s experience of SMS4dads. A final open-ended question in both interviews asked for additional feedback on their experience.

### Psychological distress

Participants’ baseline levels of distress were assessed by the Kessler K6, a brief six-question screening tool for mental illness in general populations [30]. Participants reported on a five-point Likert scale (ranging from ‘*None of the time*’ to ‘*All of the time*’) on the frequency of their feelings in regard to six factors representing their mental health during the previous 30 days. The maximum total score for the K6 is 30. Scores >13 indicate that a person is likely to be experiencing a mental health disorder, whereas scores ≥20 indicate that a person is severely unwell [32].

### Analysis

Descriptive statistics were used to investigate population characteristics (age, location, fatherhood status, distress level) and the source of recruitment, weeks receiving messages and Mood Tracker responses. Several regression models were used to assess the effect of K6 Psychological Distress Scale (dichotomized as 0–13 (no/low distress) and 13+ (clinical levels of psychological distress)), age (years), and socio-economic status (as measured by the SEIFA index). A Cox Proportional Hazards Regression model was utilized to analyse time from study entry (date of registration) to dropout (weeks). Time to dropout was defined as the time from first notification sent to the time of STOP message being sent. Participants were censored at their last received message if they did not send a STOP text. Poisson regression was utilized to analyse the number of links clicked, offset by the number of links received. A longitudinal ordinal response mixed model was utilized to analyse the mood tracking scores of participants over the course of the study; time was treated as a continuous covariate to estimate the trend. A random participant-level intercept modelled the serial correlations. We also assessed if the trend in mood differed depending on baseline K6 status through including an interaction term between K6 and time. Hazard ratios (HR), Incident rate ratios (IRR) and odds ratios (OR) are reported respectively for these regression models with 95% confidence intervals and Wald *p*-values. All analysis utilised complete case data (the mixed model used all available outcome data) under the missing at random assumption.

Statistical analyses were programmed using SAS v9.4 (SAS Institute, Cary, North Carolina, USA).

## Results

### Recruitment

During the eight-month recruitment period, 520 fathers (*M*_Age_ = 33, *SD* = 5, Range = 16–55) were enrolled from across Australia. The relative socio-economic status of fathers in this study was similar to the national average, based on Socio-economic Indexes for Areas (SEIFA) scores (M = 1007.07, SD = 64.88). The majority (66%) enrolled before the birth and most fathers heard about the program through antenatal clinics (29.30%) followed by child and family health nurses (15%), and word of mouth via a partner (13.7%) or friend (10.4%). Recruitment via GP (9.9%) and Facebook (9.7%) were more frequent than through Ultrasound clinics (1.6%), NICU (0.4%), and radio (0.4%).

At registration, participants’ mean K6 score was 11.02 (SD = 3.74). Scores greater than 13, indicating psychological distress, were reported by 112 (21.5%) of the fathers. Of those who reported K6 scores greater than 13, 18.8% (*n* = 98) had scores consistent with moderate to high levels of distress (M = 15.86), and 2.7% (*n* = 14) reported K6 scores of 20 or above (M = 22.21), indicating a severely unwell mental health status. The highest reported score was at the maximum K6 score of 30.

### Duration of participation

The average duration of participation for all fathers, including those that opted out was 21.41 weeks (SD = 10.25), ranging from 0 to 44.9 weeks. There were 78 participants (15%) who requested to leave the study by replying ‘STOP’ (*n* = 62 antenatal, *n* = 16 postnatal registration). The average number of weeks that these fathers participated before exiting was 10.94 weeks (SD = 9.55), ranging from 0 to 42.2 weeks.

A voluntary post survey completed by 36 of the 78 participants that opted out indicated that the messages were ‘not helpful’ (*n* = 11), that their ‘situation has changed’ (*n* = 9), for ‘other reasons’ (*n* = 9), they were ‘too busy’ (*n* = 5), or that they ‘did not want to do it in the first place’ (*n* = 2). Two participants reported that the messages were not helpful and indicated that they already knew the content.

**Fig. 1:**
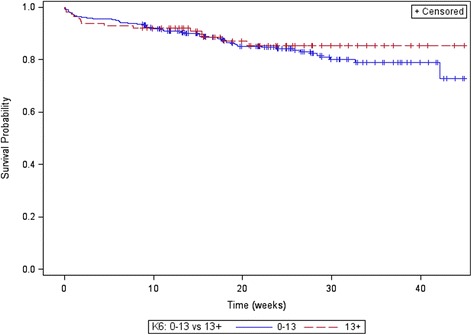
Product-limit survival estimates K6

**Fig. 2:**
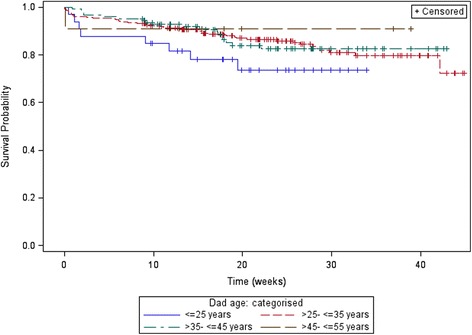
Product-limit survival estimates age categorised

The survival distributions for time to drop out stratified by K6 (0–13 and 13+), and age of the father (in 10 year age brackets) are displayed in Figs. 1 and 2. Table 2 displays the Hazard Ratios for dropout with the K6, age of the father (categorised), and SEIFA (scaled to per 100 units); while younger dads aged <25 years, lower SEIFA and lower K6 appeared to be dropping out sooner, none of these effects reached statistical significance.

**Table 2:** Hazard ratios from the Cox regression of drop out on K6, age (categorised) and SEIFA

Variable	Hazard Ratio (95% CI)	*P*-value
Dad Age: <=25	Ref	0.2496
Dad age: >25- < =35	0.53 (0.25, 1.13)	
Dad age: >35	0.54 (0.23, 1.24)	
K6 Score: 0–13	Ref	0.4825
K6 Score: 13+	0.81 (0.45, 1.46)	
SEIFA Score (100 unit)	0.79 (0.56, 1.12)	0.1838

### Use of embedded links

During the period from 29 to 10-2015 to 19–09-2016, a total of 14,118 links were distributed to the fathers.. Of the 520 participating fathers, 63.1% (*n* = 328) clicked on at least one of the links provided. The links with the highest click rates relative to how many times each individual link was sent were Kidsafe NSW ‘home safety checklist’ at a 72.7% click rate, Better Health’s ‘newborn screening’ link at a 57.1% click rate, and the ‘alcohol pregnancy partner support’ link from British Columbia’s Centre of Excellence for Women’s Health at a 50% click rate. The Kidsafe NSW ‘home safety checklist’ link had almost twice as many clicks than the number of fathers who clicked it, indicating multiple clicks per father. The Raising Children Network’s ‘dads bonding video’ was repeated in the SMS4dads messages at −16, −12, 0 (or expected birth week) and 9 weeks. This link was clicked 111 times by 68 fathers suggesting that some fathers viewed the resource multiple times. Interestingly, the length of time between receiving the links and the fathers clicking on the clicks was 2.11 days on average (SD = 3.94), ranging from 0 days to 38.3 days.

Table 3 displays the results of the regression of number of links clicked on age of the father (categorized into <=25, 25- < =35, and > 35), K6 and SEIFA. The K6 score was not associated with a difference in the rate of clicked links, however the effect for age (*p* = 0005) and SEIFA score (*p* = 0.0284) were significant. Compared to fathers aged 25 and younger, fathers aged 25–35 were estimated to have a 57% increase in their click count, and fathers aged 35 and over estimated to have a 44% increase. Every 100 unit increase in SEIFA score predicted a 9% increase in the click count.

**Table 3:** Results from the Poisson regression for number of links clicked

Variable	Estimate (95% CI)	*P*-value
Dad Age: <=25	Ref	0.0005
Dad age: >25- < =35	1.57 (1.22, 2.03)	
Dad age: >35	1.44 (1.11, 1.89)	
K6 Score: 0–13	Ref	0.4540
K6 Score: 13+	0.95 (0.85, 1.08)	
SEIFA Score (100 units)	1.09 (1.01, 1.18)	0.0237

#### Mood tracker

The mood tracker messages received a response rate of 20.5% (768/3748).’ Most fathers replied ‘Cool’ or ‘Awesome’ (70.8%), 62 (8.1%) selected ‘Shaky’, and 14 (1.8%) selected the ‘Bad’ response option. Two of those selecting ‘bad’ agreed to be contacted by the national perinatal help line. See Table 4 for a response overview.

**Table 4:** Mood tracker (MT) response overview (*N* = 506)

MT response rate	n (%)
Total MT sent	3748
Responses to MT	768 (20.5%)
Participants responded	249 (49.2%)

Table 5 displays the results of the ordinal response longitudinal analysis of the mood tracking scores from 240 participants over time. The probability of reporting worse mood scores decreased over time (OR = 0.95, *p* = 0.0295). Participants who scored 13 or greater on the K6 at baseline had increased odds (OR = 2.79, *p* = 0.0004) of scoring higher on the mood tracking score (i.e. of having worse mood tracking scores), compared to those with baseline K6 scores of 0–13. The difference in mood trends over time between those with baseline K6 13+ vs K6 < 13 was not statistically significant (interaction OR = 0.97,*p* = 0.6459).

**Table 5:** Mood trajectory analysis: ordinal regression with random person effect (*N* = 247)

Variable	Odds Ratio^a^ (95% CI)	*P*-value
Dad Age: <=25	Ref	0.6341
Dad age: >25- < =35	0.54 (0.15, 1.91)	
Dad age: >35	0.55 (0.15, 2.06)	
K6 Score: 0–13	Ref	0.0004
K6 Score: 13+	2.71 (1.56, 4.71)	
SEIFA Score (100 units)	1.04 (0.72, 1.50)	0.8412
Time	0.95 (0.91, 0.99)	0.0295

^a^Modelling probability of a HIGHER (worse) mood tracking score

### Post-study interview

Upon completion of the study, fathers were invited to complete a brief survey about their experience with SMS4dads. A total of 101 fathers completed the survey (see Table 6). When asked about the frequency of messages, 89.3% of fathers rated the frequency as good, 9.5% as not often enough, and 1.2% as too often. For 83.7% their new baby was their first child. Regarding their current financial situation, 2.2% of fathers reported being ‘very poor’ or ‘poor’, 23.9% reported to be ‘just getting by’, and 73.9% reported to be ‘reasonably comfortable’ or ‘very comfortable’. No fathers reported their financial situation as ‘prosperous’.

**Table 6:** How helpful participants found SMS4dads *N* = 101 (not all fathers answered all questions)

Question	Strongly disagree n (%)	Disagree n (%)	Not sure n (%)	Agree n (%)	Strongly agree n (%)
Being part of SMS4dads has helped me to feel less isolated as a new father.	1 (1.2%)	2 (2.4%)	11 (13.1%)	58 (69%)	12(14.3%)
SMS4dads was helpful to me in my recent experience of becoming a new dad.	0 (0%)	2 (2.4%)	4 (4.8%)	59 (70.2%)	19 (22.6%)
The messages helped me to develop a strong relationship with our new child.	0 (0%)	3 (3.6%)	26 (31%)	45 (53%)	10 (11.9%)
The messages were helpful in my relationship with my partner.	0 (0%)	1 (1.2%)	15 (17.9%)	54 (64.3%)	14 (16.7%)
The mood tracker, where I could respond to questions about how I was travelling, was useful for me.	0 (0%)	7 (8.3%)	34 (40.5%)	33 (39.3%)	10 (11.9%)

The post-study survey included the K6 again, which was completed by 90 fathers with no significant change from the mean score at the commencement of the study (*M* = 11).

### Fathers’ evaluation of messages

## Discussion

The SMS4Dads study was the first of its kind to deliver a tailored intervention to support males with the challenges and stresses they potentially faced through the antenatal and postnatal period. The primary aims of this study were to determine whether the SMS4Dads program was feasible and acceptable to fathers, both in terms of delivery method and content. In short, SMS4Dads was considered by project participants to be a useful tool for supporting their transition to fatherhood.

Fathers enrolled for the SMS4Dads study in high numbers (520 eligible participants over the eight-month recruitment period; 65 per month), providing initial evidence that a program designed to enhance the mental health and wellbeing of fathers was of interest to the intended target population. The high proportion of first time fathers suggested by the post program survey suggests that this type of support may be most relevant to men who are new to fathering. Of note was that antenatal clinics reported twice the rate of recruitment to the study than other recruitment sources (including partner referral), highlighting this setting as key in engaging fathers for programs of this nature. In Australia, antenatal clinics provide care for women throughout their pregnancy, often conducted at publicly-funded general hospitals, or private obstetrician offices. These visits create the opportunity for a range of biological, imaging, psychological, and lifestyle-related assessments, focused on the pregnant woman, and ensuring cultural safety and involving family members as per the woman’s preference [33]. A difficulty with assessing social media uptake was the imprecise assessment of the path leading fathers to enrol. Mothers may have learned of SMS4dads through Facebook and encouraged their partner to join. At baseline, around one-fifth of males self-reported levels of moderate-high psychological distress, providing further evidence that SMS4Dads had genuine potential to fill an unmet need for them in considering their roles as fathers to newborn children.

Uptake of the SMS4Dads program was also high following enrolment, with only 15% of fathers choosing to opt out of receiving the SMS messages. On average, fathers engaged with the SMS4Dads program for almost 6 months, and received around 84 messages of support during this time period. No sociodemographic or distress-related characteristic was significantly associated with retention in the SMS4Dads program, indicating the broad appeal of this approach across traditionally disadvantaged groups. These patterns of uptake of the program indicate that the pace and frequency of message provision was appropriate, with only 1% of the sample indicating that the volume of messages received was too great.

Engagement with the SMS4Dads program content was also high, with two-thirds of participants accessing additional information over and above what was provided in the SMS messages, most often within 2 days of receiving the message. Older fathers (25 years and over) did this significantly more often than did younger fathers, as did those in a reporting higher socio-economic status. This is consistent with access patterns in other areas of mental health service provision, be it for antidepressant medication (e.g., [34]), outpatient mental health clinics (e.g., [35]), or digital interventions (e.g., [36]). In particular, participants clicked on links provided in the SMS messages to retrieve more detailed information about home safety, newborn screening, and alcohol pregnancy partner support. The video focused specifically on facilitating the father-newborn bond was the most utilised resource. The practical nature of home safety may accord with fathers’ previous use of the web and the immediacy of newborn screening may have prompted this topic to be selected. Maternal alcohol consumption, reported at almost 50% during pregnancy [37] may have prompted fathers to seek further information. Father-infant bonding was also the most popular topic out of seven fathering topics offered to 137 fathers in a previous study (sex after the birth was least popular) [38].

A mood tracker feature was built into the SMS4Dads program, expressly to provide focussed, real-time screening and brief intervention for low mood during the antenatal and postnatal periods. This was a very basic form of ecological momentary assessment (EMA) and intervention (EMI), designed to capture how fathers are coping in real time during the early postnatal period. Close to half of the sample provided EMAs via the mood tracker during the study period. Higher baseline psychiatric distress corresponded with lower mood tracker scores during the intervention period, indicating the potential external validity of the mood tracker tool. Qualitative support was also provided for the mood tracker component of the SMS4Dads program, with around half of the participants reporting that the tool was useful. This is consistent with previous research indicating that people are generally receptive to EMA/EMI, especially when delivered via their mobile device, and report this experience as credible and satisfying [39]. In addition, the odds of reporting lower mood scores via the mood tracker decreased over time, indicating the potential for the mood tracker tool as an EMI. This is an important finding, given that only two out of the 14 participants who indicated ‘bad’ mood on the mood tracker took the follow-up advice to seek additional support for their mood at that point in time. The distinction between finding information or prompts to reflect ‘useful’ and actively seeking help by selecting ‘bad’ on the mood tracker and then accepting a telephone assistance may reflect the general reluctance of men to actively seek assistance [40]. Future research using SMS4Dads and similar programs might consider enhancing the intervention component of the mood tracker tool to include ‘real time’ tips on how to improve low mood (delivered as an EMI when fathers rate their mood as ‘bad’ or ‘shaky’ in the current study), to further enhance the potential mood effects of the tracking tool. This approach would also capitalise on the ‘teachable moment’ created when fathers stop and reflect on their mood, providing the opportunity to expand on this to promote active coping at times of low mood. Strategies that encourage behavioural activation and/or cognitive behavioural tools would fit well into this model, and have been translated with success to technology-based delivery in other contexts (e.g., [41]). Inclusion of these practical, just-in-time techniques may also increase the perceived effectiveness of the mood tracker tool by fathers.

The impact of the SMS4Dads program was positive, as reflected in the evaluation reported by fathers at the follow-up assessment. The most commonly endorsed benefits of the SMS4Dads program were lowered feelings of social isolation, help in transitioning to their role as a new dad, and assistance in their relationship with their partners. It is possible, given the level of financial comfort among these men enrolled in the study, that these issues came to the fore when family necessities are assured. Although few responses were received from those dropping out early, for some dads the messages were clearly not helpful, possibly because they faced more tangible difficulties. However, it may also be appropriate to develop tailored mobile phone support for identified populations of fathers who have specific needs, for example with infants in Neonatal Intensive Care [42]. To a lesser extent, participants felt that the program also helped them to develop a strong relationship with their new child even though father-infant messages comprised almost a third of messages and use of the baby’s voice (see an example in Table 1 week 2) was intended to encourage fathers to form an emotional bond with their newborns. Perhaps a more detailed evaluation procedure would discover impacts from these features on the father-infant relationship. It is interesting that these self-reported improvements and benefits did not necessarily translate to significant reductions in psychological distress among the participants from baseline to follow-up assessment. Future studies will need to employ more precise, validated measures to assess the efficacy of SMS4dads with new fathers.

### Limitations

Two significant limitations are important to note. Firstly, this was a feasibility study, and not a randomised controlled trial of the efficacy of SMS4Dads relative to other support programs, information provision, or other comparison groups. Now that the delivery modality and content of SMS4Dads has been endorsed, it is critical to carry out controlled, rigorous research evaluating the benefits of SMS4Dads, including over a long-term follow-up period. In addition, only 20% of the full sample at baseline provided follow-up assessments for the current feasibility study, and it could have been that these respondents were fathers who felt most positively and most engaged with the study program. This further points to the need to carry out a large-scale randomised trial of SMS4Dads to establish how effective it is for men transitioning through the antenatal and postnatal periods with their families.

## Conclusions

The SMS4Dads pilot study has provided evidence that a series of SMS messages, delivered over time, directly to fathers in the antenatal and postnatal periods, is a feasible and effective way of engaging them with relevant information to assist their transition to fatherhood. There are several lines of investigation that follow. Which fathers respond to which configurations of messages? Can tailoring improve fathers’ response to the mood tracker and use of links? Would a randomised controlled trial demonstrate the efficacy of SMS4dads?.
